# Limited training and transfer effects in older and young adults who participated in 12 sessions of process-based working memory training. A three-armed pretest-posttest design study

**DOI:** 10.1186/s13104-024-06844-2

**Published:** 2024-06-28

**Authors:** Ludmiła Zając-Lamparska

**Affiliations:** Faculty of Psychology, Kazimierz Wielki University, Staffa 1, Bydgoszcz, 85-867 Poland

**Keywords:** Cognitive training, Process-based training, Transfer, Working memory, Cognitive aging

## Abstract

**Objective:**

Numerous studies confirm the effectiveness of cognitive training in older adults. However, there is limited evidence of the transfer occurrence. The part of the study presented here tested the effect of 12 process-based working memory training sessions on the performance of the trained task (training effect) and other cognitive tasks (transfer effect). A pretest-posttest study design with one experimental group and two control (passive and active) groups. The sample comprised three groups of older adults: experimental (*n* = 25), passive control (*n* = 22), active control (*n* = 7), and young adults: experimental (*n* = 25), passive control (*n* = 25), and active control (*n* = 12). The study was registered after completion with a ClinicalTrials.gov Identifier: NCT06235840 on 31 January 2024.

**Results:**

Under the influence of training, the performance of the trained task improved significantly, but only in young adults. Transfer of WM training effects was not revealed. Among young adults, a testing effect was observed for the indicator of attentional focus and psychomotor speed. Moreover, the obtained results suggest the transfer from practice in multi-domain training, implemented in the active control group, to tasks that require the use of fluid intelligence. However, this finding should be interpreted with great caution due to the small size of active control groups.

**Supplementary Information:**

The online version contains supplementary material available at 10.1186/s13104-024-06844-2.

## Introduction

Numerous studies have shown that cognitive training is effective among cognitively healthy older individuals. At the same time, small to moderate effects are usually obtained and not for all indicators of cognitive functioning considered [1–6]. One of the issues crucial for the field of study on cognitive training effectiveness is the problem of cognitive transfer. The generalization of skills across similar cognitive domains or different tasks involving the same domain is known as near transfer. In contrast, the generalization across domains that are very weakly related (or even unrelated) indicates far transfer [7–9]. In this context, researchers are particularly interested in the effects of process-based training, which is based on the assumption that cognitive functions can be improved by repeatedly exercising the core processes, like working memory (WM), attentional control, or executive functions (EF), underlying them [8–11]. So far, the effectiveness of this type of training has been tested in numerous studies. However, even the results of meta-analyses have been inconclusive and contentious [12–15]. Concerning the age group of older adults, meta-analyses on the transfer of the effects of WM training have consistently shown that transfer, though possible, is limited [10, 16], and the following pattern would appear to be reproducible: (a) large training effect (improvement in the performance of trained tasks); (b) small or no near transfer effect; (c) no effect or eventually very little effect of long-distance transfer [17, 18].

In a study in which we tested the effect of WM training on electrophysiological brain activity (National Science Centre, Poland, grant no 2017/25/B/HS6/00360), we also examined the magnitude of improvement in the trained task (training effect) and changes under the influence of training in the performance of non-trained cognitive tasks with varying degrees of cognitive similarity to the trained task (near and far transfer effects). This part of the results is presented in this article. The full study was registered after completion with a ClinicalTrials.gov Identifier: NCT06235840 on 31 January 2024. Results on brain activity accompanying WM involvement in younger and older adults collected in a pretest measurement have been published [19], and a report on the impact of WM training on brain activity is in preparation.

## Method

A pretest-posttest study design was applied with one experimental group and two control (passive and active control) groups. We defined the active control group as receiving an alternative intervention comparable to the experimental group but not involving the WM training of interest [20, 21]. In the study, young adults were included in addition to older participants to allow comparisons of training effectiveness according to age.

Selection for the sample was voluntary. Participants registered in response to advertisements distributed online and on public transport in Bydgoszcz (Poland). At the same time, participants had to meet the following inclusion criteria: (1) age: 60–75 years old or 20–35 years old, where the 60–75 age group is classified as early late-adulthood, and the 20–35 group as early adulthood [22]; (2) no mental illness and neurological disorders (verified by structured interview and the Mini International Neuropsychiatric Interview – M.I.N.I. 7.0 [23]; (3) normal or corrected-to-normal vision; (4) no dementia symptoms (verified in older adults with the Polish version of the Mini-Mental State Examination, MMSE, which was purchased from the Psychological Testing Laboratory of the Polish Psychological Association, which holds the rights to the MMSE on the Polish market.) [24]; (5) signing an informed consent to participate in the study (after familiarizing oneself with the aim of the study and the conditions of participation, as well as having received satisfactory answers to all questions). The study sample comprised 116 participants including (a) older adults, divided into three research groups: experimental (E; *n* = 25; 22 women; age: *M* = 66.40, *SD* = 3.55), passive control (PC; *n* = 22; 16 women; age: *M* = 66.77, *SD* = 4.42), active control (AC; *n* = 7; 6 women; age: *M* = 65.29, *SD* = 3.35), and (b) young adults, also divided into three research groups experimental (*n* = 25; 15 women; age: *M* = 26.48, *SD* = 5.13), passive control (*n* = 25; 15 women; age: *M* = 25.48, *SD* = 4.81), active control (*n* = 12; 11 women; age: *M* = 23.92, *SD* = 5.23).

In the experimental group, participants took part in process-based adaptive training using an n-back task. In the active control group, non-adaptive multi-domain training was introduced. In both experimental and active control groups, participants were involved in 12 sessions, three in each of the four weeks. Each session lasted approximately 45 min. The passive control group was the no-contact group.

The n-back task involves WM in terms of its content updating [25, 26]. This task is based on the continuous presentation of items (letters in this study) that appear and disappear one by one. During each presentation, the participant must judge whether the currently displayed item matches the item presented ‘n’ trials earlier [27]. The n-back task used in the current study was computerized and was programmed in *PsychoPy* software [28]. All participants started their training from the 1-back level. The training was adaptive, meaning participants progressed to higher (or dropped to lower) difficulty levels according to the performance accuracy achieved. The increasing/decreasing difficulty was based on a rise/fall in the ‘n’ parameter.

In the active control group, non-adaptive multi-domain computerized training was introduced, which included tasks involving visual-spatial functions, visual and verbal memory, analogical reasoning on visual material, deductive reasoning on verbal material, and calculia.

The assignment to the research groups was not random and was based on an n-back task and Raven’s Standard Progressive Matrices performance because the aim was to equalize the cognitive functioning of individual research groups (within age groups).

A series of measures of cognitive functioning were used in the pretest and posttest: (a) the n-back task at three levels of difficulty, i.e. 1-back, 2-back, and 3-back, in which the sensitivity index (d’) was used as the indicator of task performance; d’ was calculated with the formula for yes/no tasks: d’ = z(H) – z(FA), where H and FA are the Hits and False Alarm rates, respectively, and z(H) and z(FA) are the z-transformations [29]; (b) computerized OperationSpan Task (OSPAN) [30]– to assess WM capacity; (c) two subtests from the Wechsler Adult Intelligence Scale – WAIS-R(PL) [31]: the Digit Span Subtest (Dig-Span) to measure short-term memory and WM capacity, and The Digit-Symbol Coding Subtest (Dig-Symb) to measure attentional focus and psychomotor speed; (d) Raven’s Standard Progressive Matrices in Polish standardization (TMS) [32] to measure fluid intelligence, the raw score was used as the performance indicator, 50% of the sample performed the classic version in the pretest and the parallel version in the posttest, while 50% did the opposite.

## Results

**Fig. 1:**
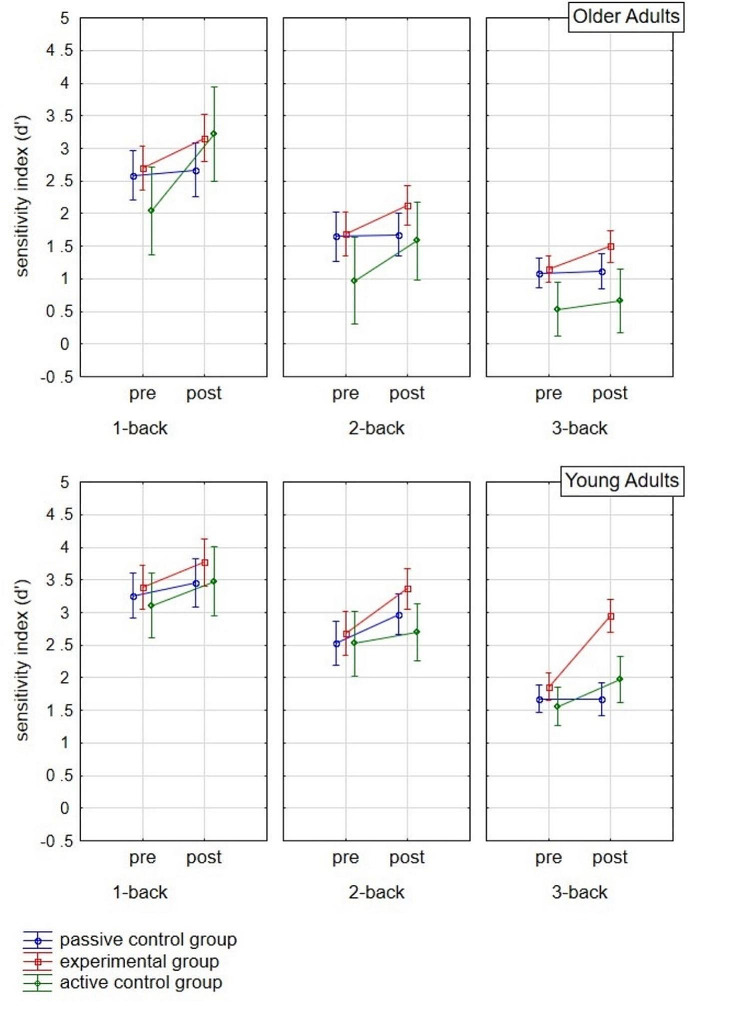
Performance of the n-back task (d’) at three difficulty levels in the initial and final measurements in the experimental, passive control, and active control groups of older and young adults

To assess the effect of training on the performance of the trained task (n-back), a mixed-design ANOVA was conducted, with two within-person factors, i.e., repeated measurement (pretest-posttest) and the n-back task difficulty level, and two between-person factors: research group and age group (Fig. 1). As expected, the effect of the age group proved to be significant (*F* = 73.51; *p* < .001; *η*^*2*^*p* = 0.42), with poorer performance in the older adult group. No significant interaction effect of age group and research group was observed (*F* = 0.48; *p* = .623; *η*^*2*^*p* = 0.01). Also in line with expectations, significant was the effect of the n-back task difficulty level (*F* = 222.58; *p* < .001; *η*^*2*^*p* = 0.69), with decreasing levels of performance at increasing difficulty. At the same time, there was a significant interaction effect of the task difficulty with the age group (*F* = 5.50; *p* = .005; *η*^*2*^*p* = 0.05) but not with the research group (*F* = 1.43; *p* = .238; *η*^*2*^*p* = 0.03). The main effect of repeated measurement was found to be statistically significant (*F* = 38.09; *p* < .001; *η*^*2*^*p* = 0.28), as well as the interaction of repeated measurement with the research group (*F* = 6.65; *p* = .002; *η*^*2*^*p* = 0.12) but not with the age group (*F* = 0.16; *p* = .694; *η*^*2*^*p* = 0.002). The interaction effect of the three factors, i.e., repeated measurement, age group, and research group, was not statistically significant (*F* = 1.39; *p* = .191; *η*^*2*^*p* = 0.03). However, the interaction effect of the four factors, i.e., the three mentioned above plus the n-back task difficulty level, did (*F* = 2.60; *p* = .037; *η*^*2*^*p* = 0.05).

In further analyses, a series of ANOVA was performed for each cognitive measure considered in the study, including three levels of the n-back task. For the results collected in the pretest and posttest (separately), the main effects of age group, research group, and effect of their interaction were analysed. Furthermore, the effect of repeated measurement (pretest vs. posttest) and its interaction with age group, research group, and both of those factors were tested. Each ANOVA was supplemented by post hoc analysis using the Tukey-Kramer test. The results of the series of ANOVA are presented in Table 1, while the means and standard deviations of each cognitive measure at the pretest and posttest for each age and research group are presented in Table 2. The results of the post hoc analysis are reported in the text, when appropriate, as a supplement to the ANOVA results.

**Table 1:** Mixed-design ANOVA results for pretest, posttest, and repeated measurement for trained and not-trained cognitive tasks performance

Cognitive Measure/ Effect	Pretest			Posttest			Pretest vs. Posttest		
	F	*p*	η^2^*p*	F	*p*	η^2^*p*	F	*P*	η^2^*p*
1-back d’									
age gr.	21.53	**< 0.001**	0.17	9.15	**0.003**	0.08	18.99	**< 0.001**	0.15
res. gr.	2.09	0.129	0.04	2.06	0.132	0.04	2.06	0.133	0.04
age gr.* res. gr.	0.18	0.836	0.003	0.77	0.466	0.01	0.13	0.876	0.00
pre-post							21.68	**< 0.001**	0.17
pre-post*age gr.							1.80	0.182	0.02
pre-post*res. gr.							2.79	0.066	0.05
pre-post*res. gr.*age gr.							1.46	0.238	0.03
2-back d’									
age gr.	36.36	**< 0.001**	0.25	55.88	**< 0.001**	0.34	64.09	**< 0.001**	0.38
res. gr.	2.22	0.114	0.04	5.31	**0.006**	0.09	4.80	**0.010**	0.08
age gr.* res. gr.	0.45	0.638	0.01	0.04	0.960	0.00	0.16	0.850	0.00
pre-post							19.95	**< 0.001**	0.16
pre-post*age gr.							0.34	0.562	0.00
pre-post*res. gr.							1.46	0.236	0.03
pre-post*res. gr.*age gr.							0.85	0.428	0.02
3-back d’									
age gr.	52.37	**< 0.001**	0.33	69.73	**< 0.001**	0.39	82.77	**< 0.001**	0.44
res. gr.	4.83	**0.010**	0.08	26.92	**< 0.001**	0.33	16.91	**< 0.001**	0.25
age gr.* res. gr.	1.21	0.303	0.02	7.73	**< 0.001**	0.13	4.30	**0.016**	0.08
pre-post							33.70	**< 0.001**	0.24
pre-post*age gr.							6.22	**0.014**	0.06
pre-post*res. gr.							17.62	**< 0.001**	0.25
pre-post*res. gr.*age gr.							6.19	**0.003**	0.11
OSPAN									
age gr.	34.48	**< 0.001**	0.25	21.07	**< 0.001**	0.16	33.01	**< 0.001**	0.24
res. gr.	0.15	0.866	0.003	0.52	0.598	0.01	0.11	0.894	0.00
age gr.* res. gr.	0.48	0.620	0.01	2.09	0.129	0.04	1.11	0.334	0.02
pre-post							2.31	0.132	0.02
pre-post*age gr.							4.69	**0.033**	0.04
pre-post*res. gr.							1.81	0.169	0.03
pre-post*res. gr.*age gr.							0.86	0.427	0.02
Dig-Span forward									
age gr.	2.51	0.116	0.02	1.56	0.214	0.01	2.44	0.121	0.02
res. gr.	1.47	0.234	0.03	0.02	0.977	0.00	0.51	0.603	0.01
age gr.* res. gr.	1.15	0.321	0.02	1.73	0.182	0.03	1.31	0.274	0.02
pre-post							1.12	0.291	0.01
pre-post*age gr.							0.04	0.838	0.00
pre-post*res. gr.							1.36	0.260	0.02
pre-post*res. gr.*age gr.							2.15	0.121	0.04
Dig-Span backward									
age gr.	9.30	**0.003**	0.08	7.19	**0.008**	0.06	9.87	**0.002**	0.08
res. gr.	1.15	0.322	0.02	0.81	0.447	0.01	1.17	0.315	0.02
age gr.* res. gr.	0.38	0.684	0.01	1.46	0.236	0.03	0.99	376	0.02
pre-post							4.96	**0.028**	0.04
pre-post*age gr.							0.11	0.746	0.00
pre-post*res. gr.							0.03	0.973	0.00
pre-post*res. gr.*age gr.							0.72	0.492	0.01
Dig Symb									
age gr.	85.63	**< 0.001**	0.44	121.57	**< 0.001**	0.52	112.90	**< 0.001**	0.51
res. gr.	1.17	0.314	0.02	1.24	0.292	0.02	1.31	0.274	0.02
age gr.* res. gr.	0.07	0.936	0.00	0.27	0.767	0.00	0.16	0.849	0.00
pre-post							42.01	**< 0.001**	0.28
pre-post*age gr.							9.57	**0.002**	0.08
pre-post*res. gr.							0.17	0.848	0.00
pre-post*res. gr.*age gr.							0.19	0.824	0.00
TMS									
age gr.	44.47	**< 0.001**	0.29	48.16	**< 0.001**	0.31	52.24	**< 0.001**	0.32
res. gr.	1.46	0.236	0.03	0.50	0.607	0.01	0.31	0.734	0.01
age gr.* res. gr.	0.67	0.512	0.01	0.04	0.962	0.00	0.24	0.789	0.00
pre-post							26.56	**< 0.001**	0.20
pre-post*age gr.							1.54	0.218	0.01
pre-post*res. gr.							6.21	**0.003**	0.10
pre-post*res. gr.*age gr.							1.45	0.239	0.03

Note d’ – sensitivity index; OSPAN – Operation Span task; Dig-Span - the Digit Span Subtest from the Wechsler Adult Intelligence Scale; Dig-Symb – the Digit-Symbol Coding Subtest from the Wechsler Adult Intelligence Scale; TMS – Raven’s Standard Progressive Matrices in Polish standardization; age gr. – age group; res. gr. – research group; pre-post – repeated measurement

**Table 2:** The means and standard deviations of the scores of the particular cognitive measures in each research group (experimental, passive control, and active control) and age group (older and young adults)

Cognitive measure	Research group	Older Adults		Young Adults	
		Pretest *M (SD)*	Posttest *M (SD)*	Pretest *M (SD)*	Posttest *M (SD)*
1-back d’	E	2.69 (0.72)	3.15 (1.06)	3.37 (0.65)	3.77 (0.64)
	PC	2.54 (0.96)	2.680 (1.25)	3.272 (0.74)	3.50 (0.42)
	AC	2.10 (1.49)	3.16 (1.05)	3.05 (0.51)	3.379 (0.51)
2-back d’	E	1.69 (0.79)	2.12 (0.90)	2.69 (0.74)	3.34 (0.75)
	PC	1.69 (0.65)	1.76 (0.56)	2.53 (0.74)	2.96 (0.75)
	AC	1.08 (1.38)	1.58 (0.67)	2.35 (1.08)	2.69 (0.47)
3-back d’	E	1.14 (0.42)	1.50 (0.50)	1.82 (0.61)	2.96 (0.92)
	PC	1.10 (0.39)	1.17 (0.42)	1.68 (0.48)	1.67 (0.42)
	AC	0.55 (0.68)	0.76 (0.60)	1.56 (0.44)	1.92 (0.50)
OSpan	E	0.63 (0.17)	0.67 (0.13)	0.78 (0.13)	0.75 (0.16)
	PC	0.61 (0.17)	0.69 (0.16)	0.78 (0.11)	0.79 (0.10)
	AC	0.60 (0.25)	0.60 (0.22)	0.83 (0.10)	0.83 (0.08)
Dig-Span forward	E	5.00 (1.26)	5.44 (1.04)	6.12 (1.74)	6.68 (2.17)
	PC	5.55 (1.26)	6.14 (2.03)	6.32 (2.14)	6.000 (2.00)
	AC	6.43 (1.90)	6.00 (1.92)	6.17 (1.47)	6.33 (1.17)
Dig-Span backward	E	4.60 (1.23)	4.80 (0.91)	6.08 (1.63)	6.64 (2.22)
	PC	5.09 (1.69)	5.50 (1.71)	5.96 (2.19)	6.28 (2.05)
	AC	5.57 (0.98)	6.14 (1.68)	6.58 (2.35)	6.58 (2.11)
Dig-Symb	E	41.36 (8.66)	44.52 (9.52)	60.52 (10.97)	66.72 (11.69)
	PC	38.46 (12.49)	40.32 (11.18)	59.08 (8.63)	65.44 (8.06)
	AC	42.71 (12.35)	44.29 (9.45)	63.17 (10.59)	69.25 (13.13)
TMS	E	44.72 (7.79)	45.52 (7.52)	53.16 (5.99)	54.79 (4.99)
	PC	42.32 (9.54)	44.68 (7.77)	54.24 (5.60)	54.12 (4.89)
	AC	40.14 (11.52)	46.86 (7.54)	50.75 (5.03)	55.33 (3.55)

Note d’ – sensitivity index; OSPAN – Operation Span task; Dig-Span – the Digit Span Subtest from the Wechsler Adult Intelligence Scale; Dig-Symb – the Digit-Symbol Coding Subtest from the Wechsler Adult Intelligence Scale; TMS – Raven’s Standard Progressive Matrices in Polish standardization; E – experimental group; PC – passive control group; AC – active control group

According to the results, older adults show significantly worse cognitive performance than young adults in the pretest and posttest for all cognitive measures, except for the Digit Span Forward task, which involves short-term memory.

At the pretest, the research groups do not differ significantly in cognitive functioning. Only in the 3-back task did the study group effect prove to be statistically significant, but post hoc analysis revealed no significant differences between research groups in either older (E vs. PC *p* = 1.00; E vs. AC *p* = .223; PC vs. AC *p* = .296) or young adults (E vs. PC *p* = .912; E vs. AC *p* = .811; PC vs. AC *p* = .993).

Significant changes between the initial and final measurements were observed on several cognitive measures (Table 1). At the same time, a significant interaction effect of repeated measurement with the research group was revealed only for the trained 3-back task (where, in addition, the interaction effect of three factors: repeated measurement, research group, and age group was significant) and for the untrained TMS test. Moreover, in the case of the 3-back task, the effects of the research group and the interaction of the research group and age were significant in the posttest, which was not observed for the TMS test. Post hoc analysis revealed that significant improvements in performance on the 3-back task occurred only in the experimental group of young adults (*p* < .001), not in the other research groups of young adults (PC *p* = 1.00; AC *p* = .446) and older adults (E *p* = .136; PC *p* = 1.00; AC *p* = .998). As for differences between the research groups in the posttest, among young adults, the experimental group performed significantly higher than the passive control (*p* < .001) and active control (*p* < .001) groups, which, in turn, did not differ from each other (*p* = .912). This result was not replicated in older adults, where there was no significant difference between the research groups in the posttest (E vs. PC *p* = .467; E vs. AC *p* = .188; PC vs. AC *p* = .787). Therefore, based on the results obtained, it can be concluded that the performance of the 3-back task improved under the influence of training, but only in young adults. In contrast, for the TMS test, a significant improvement was revealed by post hoc analysis only in the active control group of older adults (*p* = .023), not in the other research groups of older adults (E *p* = 1.00; PC *p* = .518), and any research group of young participants, although in the passive control group of young adults the difference reached the level of statistical trend (E *p* = .915; PC *p* = 1.00; AC *p* = .069). Thus, in the case of the TMS test, we have a rationale for inferring a near transfer from multi-domain training (covering tasks involving analogical reasoning) to the performance of a test measuring fluid intelligence, primarily in older adults.

In contrast, for the 1-back, 2-back, and Digit Span Backward tasks, only the effect of repeated measurement was significant, with no interaction with the study group. Such a result reflects the testing effect (test-enhanced learning) rather than the impact of training. At the same time, while the research group effect was statistically significant in the 2-back task in the posttest, the post hoc analysis did not show statistically significant differences between the research groups in older adults (E vs. PC *p* = .583; E vs. AC *p* = .796; PC vs. AC *p* = .998) or young adults (E vs. PC *p* = .442; E vs. AC *p* = .241; PC vs. AC *p* = .944). In parallel, according to the results of the post hoc analysis, a significant improvement in the performance of the 2-back task occurred only in the experimental group of young participants (*p* = .018), not in the other research groups of young adults (PC *p* = .362; AC *p* = .970) nor older adults (E *p* = .400; PC *p* = 1.00; AC *p* = .847). In the 1-back task, on the other hand, there was no significant change in the young participants (E *p* = .614; PC *p* = .994; AC *p* = .972), while the older ones showed improvement at the level of statistical trend but only in the active control group (E *p* = .260; PC *p* = 1.00; AC *p* = .059).

Furthermore, for the Digit Symbol task, significant effects of repeated measurement and the interaction of repeated measurement with age group were revealed, which may suggest the presence of an age-dependent testing effect. This interpretation is supported by the results of the post hoc analysis, which disclose a significant improvement in performance on this task in each of the research groups of young adults (E *p* < .001; PC *p <* .001; AC *p* = .044) but in none of the older adult groups (E *p* = .327; PC *p* = .960; AC *p* = 1.00).

Finally, for the OSPAN task, the interaction effect of repeated measurement with age group was found without the significance of the main effect of repeated measurement. However, post hoc analyses indicated a significant improvement in performance on this task only in the passive control group of older adults (*p* = .035) but not in the other research groups of older adults (E *p* = .897; AC *p* = 1.00) or young adults (E *p* = .921; PC *p* = 1.00; AC *p* = 1.00). This result eludes interpretation in the light of the assumptions made based on the existing knowledge and seems to bear the mark of coincidence.

## Discussion

The aim of the part of the study presented here was to test whether WM training based on an n-back task leads to improved performance on trained tasks and the transfer to other cognitive tasks among older adults and young adults.

The results indicate that the training positively affected the performance of the trained task, but only among young adults and at higher levels of task difficulty. The differences in the older adults’ experimental group were too small to prove significant in post hoc analyses. This finding is inconsistent with the conclusions of the meta-analyses presented in the introduction. However, the training implemented in the current study was relatively short, which may have influenced its effects. On the other hand, it is possible that process-based training involving WM has limited effectiveness among older adults.

Going further, we do not observe the phenomenon of the near and far transfer of practice from the n-back task to other cognitive tasks. The lack of transfer among older adults could be explained by the limited effects of the training itself. Still, transfer also did not occur in young adults, in whom training significantly improved performance on the trained n-back task. However, in young adults, there was an apparent testing effect (test-enhanced learning) for performance on a task requiring attentional focus and psychomotor speed.

Interesting appear to be the results regarding changes in performance on a test measuring fluid intelligence. This is because they suggest the transfer from practice in multi-domain training, implemented in the active control group, to tasks that require the use of fluid intelligence. Such results could be explained by the use, as a part of multi-domain training, tasks that enforce analogical reasoning and rule recognition, which are also needed when performing TMS test. Thus, it could be argued that a phenomenon of near transfer is revealed for training treated as an active control. This result, however, should be interpreted with great caution due to the small size of active control groups, especially among older adults. At the same time, it can be treated as a contribution to further research.

## Limitations

- The main limitation of the study is the unequal size of the research groups, or more precisely, the small size of the active control groups. This significantly limits the ability to conclude based on the effects obtained for these groups.
- The research and age groups are not gender balanced. Moreover, active control groups appear younger than the other two research groups (within each age group), but there are no statistically significant differences in age between the three research groups among older adults (*F* = 0.38; *p* = .683; *η*^*2*^*p* = 0.02) and young adults (*F* = 1.07; *p* = .351; *η*^*2*^*p* = 0.04).
- The baseline levels of performance of the cognitive tasks used are not perfectly equal in individual research groups (within age groups). However, as reported above, there were no statistically significant differences in the performance of tasks that were the criteria for group assignment.

### Electronic supplementary material

Below is the link to the electronic supplementary material.

Supplementary Material 1

## Data Availability

Data is provided within the manuscript or supplementary information files.The study was registered after completion of the study with a ClinicalTrials.gov Identifier: NCT06235840 on 31 January 2024.

## Abbreviations

AC: Active control group
d’: Sensitivity index
Dig-Span: The Digit Span Subtest from the Wechsler Adult Intelligence Scale
Sig-Symb: The Digit-Symbol Coding Subtest from the Wechsler Adult Intelligence Scale
E: Experimental group
EFL: Executive functions
MANOVA: Multivariate analysis of variance
M.I.N.I. 7.0: The Mini International Neuropsychiatric Interview
MMSE: The Mini-Mental State Examination
OSPAN: The Operation Span task
PC: Passive control group
T-K: The Tukey-Kramer post hoc test
TMS: Raven’s Standard Progressive Matrices in Polish Standardization
WAIS-R(PL): The Wechsler Adult Intelligence Scale
WM: Working memory
